# Salivary microbial signature highlighting actinomyces as a predictor of immune-checkpoint inhibitor monotherapy response in advanced non–small cell lung cancer

**DOI:** 10.1186/s12967-025-07570-4

**Published:** 2026-01-17

**Authors:** Silvia Cavaliere, Marta Fogolari, Michele Iuliani, Simone Foderaro, Alessio Cortellini, Sonia Simonetti, Emanuele Claudio Mingo, Silvia Calagna, Marco Russano, Bruno Vincenzi, Giuseppe Tonini, Silvia Angeletti, Francesco Pantano

**Affiliations:** 1 Medical Oncology, Fondazione Policlinico Universitario Campus Bio-Medico, Via Alvaro del Portillo 200, 00128 Roma, Italy; 2 Research Unit of Clinical Laboratory Science, Department of Medicine and Surgery, Università Campus Bio-Medico di Roma, Via Alvaro del Portillo, 21, 00128 Roma, Italy; 3 Operative Research Unit of Laboratory, Fondazione Policlinico Universitario Campus Bio-Medico, Via Alvaro del Portillo, 200, 00128 Roma, Italy; 4 Department of Medicine and Surgery, Università Campus Bio-Medico di Roma, Via Alvaro del Portillo 21, 00128 Roma, Italy

**Keywords:** Salivary microbiome, Immune-checkpoint inhibitors, Non–small cell lung cancer, Actinomyces

## Abstract

**Background:**

Immune checkpoint inhibitors (ICIs) have improved survival in advanced non-small cell lung cancer (NSCLC), yet reliable biomarkers beyond programmed death-ligand 1 (PD-L1) expression remain limited. Increasing evidence links the gut microbiome to ICI activity, but the predictive value of the salivary microbiome is poorly defined.

**Methods:**

We prospectively analyzed baseline saliva from 71 stage IV NSCLC patients treated with anti–PD-1/PD-L1 (ICI) monotherapy. After quality control, 70 samples underwent 16 S rRNA gene sequencing of the V1–V3 region. Microbial diversity, differential abundance (LEfSe, Mann-Whitney/Kruskal-Wallis with false discovery rate correction) and survival associations (Kaplan-Meier; Cox proportional-hazards with LASSO-based variable selection and 1000-fold bootstrap validation) were examined. In this cohort, an exploratory genus-level cut-off was derived by receiver operating characteristic (ROC) analysis.

**Results:**

α-diversity and β-diversity did not differ between responders (progression-free survival (PFS) ≥ 12 months; *n* = 18) and non-responders (*n* = 52). Differential‑abundance profiling revealed a graded enrichment of the phylum Actinobacteria across all lower ranks, class Actinobacteria, order Actinomycetales, family Actinomycetaceae and genus *Actinomyces*,in non‑responders (LEfSe LDA > 3.5; *p* = 0.001 for each level; FDR ≤ 0.049). ROC analysis suggested an *Actinomyces* relative abundance of 11% (AUC = 0.768; sensitivity 0.94; specificity 0.44) as a data-driven threshold, classifying patients into low (≤ 11%, *n* = 46) and high (> 11%, *n* = 24) groups. High abundance was associated with shorter PFS (median 3 vs. 4 months; HR = 2.16, 95% CI 1.21–3.88, *p* = 0.009) and overall survival (OS) (median 5 vs. 9 months; HR = 2.61, 95% CI 1.48–4.61, *p* < 0.001) after multivariable adjustment for ECOG status, treatment line, corticosteroid and opioid use, smoking, histology and metastatic sites. Bootstrap validation supported model stability, with median bootstrap HRs of 2.56 (PFS) and 2.63 (OS), with narrow percentile CIs (PFS 1.57–4.49; OS 1.40–6.34) overlapping the original estimates.

**Conclusions:**

In this exploratory cohort, salivary microbiome signature characterized by high *Actinomyces* abundance was independently associated with poorer ICI outcomes in NSCLC. Saliva profiling is non-invasive and, if validated in larger and independent cohorts, may complement tumour PD-L1 and clinical factors to refine patient stratification.

**Supplementary Information:**

The online version contains supplementary material available at 10.1186/s12967-025-07570-4.

## Introduction

Immune checkpoint inhibitors (ICIs) have revolutionized the treatment of non-small cell lung cancer (NSCLC), providing substantial survival gains in a subset of patients [1]. However, durable responses occur in only a minority of patients, underscoring an urgent need for more reliable predictive biomarkers [2]. Currently, the expression of programmed death-ligand 1 (PD-L1) on tumor cells represents the only biomarker routinely implemented in clinical practice to guide patient selection for anti–PD-1/PD-L1 ICIs. Despite its widespread use, PD-L1 exhibits substantial limitations, including variable predictive value, inter- and intra-tumoral heterogeneity, and lack of sensitivity or specificity to capture the complexity of the tumor–host immune interplay ( [3, 4]). As a result, a considerable fraction of patients with high PD-L1 expression do not respond to ICIs, while some patients with low or negative PD-L1 status experience meaningful clinical benefit. This highlights the urgent need to identify novel, more robust biomarkers to refine patient stratification and optimize therapeutic outcomes [5].

In recent years, the gut microbiota has emerged as a pivotal regulator of systemic immunity and a potential modulator of response to ICIs in various malignancies, including NSCLC. Numerous studies have demonstrated that specific gut bacterial taxa are associated with improved ICI efficacy, leading to a surge of interest in the microbiome as a biomarker and therapeutic target [6–9]. However, while the influence of the intestinal microbiota on cancer immunotherapy has been extensively investigated, the role of the salivary microbiome remains largely unexplored. This knowledge gap is particularly relevant in NSCLC, where the oral cavity and upper airways are anatomically and functionally linked to the pulmonary environment, and where the oral/salivary microbiome could provide a non-invasive window into host–microbiome–tumor interactions and ICI outcomes.

The salivary microbiota, which is a primary source of microorganisms colonizing the respiratory tract, may thus offer unique insights into the host–microbiome–tumor axis relevant to lung cancer biology and ICI outcomes [10]. Recent evidence suggests that the composition of the salivary microbiome may reflect that of the lower airways and lungs, potentially serving as a non-invasive surrogate for pulmonary microbiota profiling [11]. Investigating the salivary microbiome as a predictive biomarker could therefore address a critical unmet need in the field, enabling better patient selection and personalized immunotherapeutic strategies in NSCLC.

In this study, we evaluate the association between the composition of the salivary microbiota and clinical outcomes in advanced NSCLC patients treated with ICIs. Our primary endpoint was progression-free survival (PFS), with overall survival (OS) and ICI response as key secondary endpoints; we hypothesized that distinct baseline salivary microbiota profiles would identify patients with different clinical outcomes to ICI therapy in advanced NSCLC.

## Materials and methods

### Study design and sample collection

Seventy-one patients with stage IV NSCLC eligible for ICI monotherapy were enrolled at Fondazione Policlinico Universitario Campus Bio-Medico (Rome, Italy) between February 2019 and May 2023. The study adhered to the principles of the Helsinki Declaration. All experimental protocols received approval from the Internal Review and Ethics Boards of Fondazione Policlinico Universitario Campus Bio-Medico (Protocol No. 48.17OSS). Key eligibility criteria included age ≥ 18 years, histologically confirmed stage IV NSCLC, Eastern Cooperative Oncology Group (ECOG) performance status 0–1, adequate organ function, and planned treatment with anti–PD-1/PD-L1 ICIs (Pembrolizumab, Nivolumab, Cemiplimab, Atezolizumab) in first- or second-line settings. Exclusion criteria included recent malignancies, prior ICI treatment, immunodeficiency, ongoing infections, systemic steroids, or antibiotics within 20 days before sampling; chronic antibiotic or probiotic intake beyond this time window was not prospectively and systematically recorded and could not be evaluated in sensitivity analyses.

CT or PET/CT assessed tumor response per RECIST v1.1, and patients were categorized as responders (complete/partial response or stable disease ≥ 12 months) or non-responders. Baseline saliva samples were collected using saliva DNA collection and preservation devices (NORGEN BIOTEK, Thorold, Canada) on the day before the first ICI infusion and were stored at room temperature until processing. Each sample was assigned a unique anonymized code. Clinical data were prospectively recorded; missingness for baseline covariates was low, and time-to-event analyses were performed using complete-case summaries (i.e., including only patients with available data for all variables in a given model).

### DNA extraction and 16 S rRNA gene sequencing

Bacterial DNA was isolated using the QIAamp^®^ DNA Microbiome Kit (QIAGEN, Hilden, Germany), according to the protocol’s specifications. DNA was quantified using NanoDrop 2000 instrument (Thermo Scientific, Waltham, MA, USA) and stored at − 80 °C. Libraries were prepared using the Microbiota Solution A Kit (Arrow Diagnostics), targeting V1–V3 regions of the 16 S rRNA gene via two-step PCR. Amplicons (~ 640 bp) were verified with TapeStation (Agilent), purified (AMPure XP beads), and indexed. Sequencing was performed on an Illumina MiSeq platform (MiSeq Reagent Nano Kit v2, 500-cycles).

### Microbiome analysis

FASTQ files were processed with MicrobAT (SmartSeq). Primers were trimmed, and low-quality or short reads were filtered. Sequencing depth per sample was high and relatively homogeneous across the cohort, with all libraries meeting predefined quality and depth thresholds deemed adequate for downstream diversity and differential-abundance analyses.

Briefly, bases with Phred quality scores below a predefined threshold were removed from the 3′ end, and reads shorter than a minimum length were discarded. Sequences were clustered into operational taxonomic units (OTUs) at ≥ 97% similarity, and taxonomic assignment was performed using the MicrobAT built-in classifier against its default reference database. Downstream analyses were conducted with MicrobiomeAnalyst 2.0 after filtering low-count, low-prevalence, and low-variance features. Features with very low counts across samples and those present in only a small fraction of samples were removed to reduce sparsity and noise. Total sum scaling (TSS) was used for normalization. Zero values were retained in the normalized abundance matrix and only replaced by a small pseudocount when required for log-transformed analyses. Rarefaction was not applied, in line with current recommendations favoring normalization over subsampling for compositional microbiome data. All diversity and differential-abundance analyses were therefore performed on TSS-normalized, non-rarefied data.

Microbial diversity of the samples was assessed in the form of α-diversity and β-diversity. The Shannon index was used as the metric for calculating α-diversity, while β-diversity was evaluated using ordination methods such as Principal Coordinates Analysis (PCoA) with Bray-Curtis distance. Permutational Multivariate Analysis of Variance (PERMANOVA) was employed for statistical analysis of β-diversity patterns. Linear Discriminant Analysis Effect Size (LEfSe) was performed to identify specific taxa associated with the outcome from ICIs. This analysis uses Linear Discriminant Analysis (LDA) to quantify the effect of each discriminant taxon, allowing us to understand which microbes contribute most to the observed differences. Univariate analysis was conducted using the Mann–Whitney U and Kruskal–Wallis tests, as appropriate. False Discovery Rate (FDR) correction was applied for multiple hypothesis testing, with p-values ≤ 0.05 considered statistically significant. Negative extraction and PCR controls were included in each sequencing run and inspected to monitor potential contamination; these controls yielded negligible read counts and did not show enrichment of taxa of interest. When multiple sequencing runs were required, potential batch effects were evaluated by visual inspection of PCoA plots colored by run and by PERMANOVA; no significant clustering by sequencing run was detected.

### Definition of *Actinomyces* cut-off

The exploratory cut-off value for *Actinomyces* abundance was determined using receiver operating characteristic (ROC) curve analysis, based on the ability to discriminate responders from non-responders to ICIs. The area under the curve (AUC) was calculated, and the threshold that maximized both sensitivity and specificity (Youden’s Index) was selected as data-driven, cohort-specific threshold rather than a predefined clinical decision point. Patients were subsequently categorized as “≤11%” or “>11%” based on this threshold. The binary variable was encoded such that “≤11%” was the reference category.

### Statistical analysis

The primary endpoint of the study was PFS, defined as the time from initiation of anti–PD-1/PD-L1 therapy to radiographic disease progression or death from any cause, whichever occurred first. OS, defined as the time from treatment initiation to death from any cause, and ICI response (responders vs. non-responders) were prespecified secondary endpoints. Patients without an event at the time of analysis were censored at the date of last follow-up. Percentages are reported with one decimal place, whereas hazard ratios (HRs) and 95% confidence intervals (CIs) are reported with two decimals.

Kaplan-Meier survival analysis was performed to estimate PFS and OS for patient subgroups defined by *Actinomyces* abundance. Survival curves were generated using the Kaplan-Meier method, and differences between groups were assessed with the log-rank test. Median survival times and 95% confidence intervals (CI) were calculated for each group. The number of patients at risk at each time point was reported below the survival curves. All analyses were conducted using the “survival” and “survminer” packages in R (version 4.5.0). A two-sided p-value < 0.05 was considered statistically significant. Univariate Cox proportional hazards regression models were first performed to assess the association of each clinical and biological variable with PFS and OS, with HR and 95% CI reported for each predictor. Variable selection for the multivariable model was performed using Least Absolute Shrinkage and Selection Operator (LASSO) penalized Cox regression, as implemented in the glmnet package in R. To optimize the balance between model complexity and statistical power, we selected the regularization parameter (lambda) corresponding to a model with an appropriate number of variables relative to the number of observed events, rather than relying exclusively on the “1-standard error” rule (lambda1.se) or the minimum cross-validation error (lambda.min). Selected variables were jointly entered into a multivariable Cox regression model to estimate their independent association with PFS and OS, and the proportional hazards assumption was checked for each variable using Schoenfeld residuals. To evaluate the internal validity and robustness of the final model, bootstrap resampling with 1,000 iterations was conducted: for each bootstrap sample, HRs were re-estimated using the same multivariable model structure. The distribution of HRs across bootstrap replicates was summarized by the median and percentile-based (2.5th–97.5th) confidence intervals. All analyses were performed in R (version 4.5.0) using the survival, survminer, glmnet, and boot packages, and statistical significance was defined as a two-sided p-value < 0.05.

## Results

### Patient cohort and clinical characteristics

Seventy-one patients with advanced NSCLC were prospectively enrolled between 2019 and 2023. Following exclusion of one patient (37CA) due to low sequencing quality, 70 patients were included in the final analysis (42 males and 28 females). The median age of the study population was 70 years. A majority of patients (86%) were either current or former smokers, while 77% presented with adenocarcinoma histology. At baseline, 57% had an ECOG performance status of 1, and PD-L1 expression ≥ 50% was detected in 55.5% of cases. Based on PFS, 18 patients (25.7%) were classified as responders (PFS ≥ 12 months), whereas the remaining 52 patients (74.3%) were categorized as non-responders. A comprehensive summary of clinicopathological characteristics is provided in Table 1.

**Table 1:** Clinicopathological characteristics

Feature	*N* (%)
Age ≥70 <70	42 (60.0) 28 (40.0)
Sex Female Male	21 (30.0) 49 (70.0)
**Smoke** Never Former/Current	10 (14.3) 60 (85.7)
**ECOG-PS** 0 1	30 (42.9) 40 (57.1)
**PD-L1 Expression** ≥ 50% 1–49% < 1%	39 (55.7) 14 (20.0) 17 (24.3)
**Histology** Adenocarcinoma Squamous	54 (77.1) 16 (22.9)
**Treatment Line** First Second	35 (50.0) 35 (50.0)
**Treatment Type** Anti PD-1 Anti PD-1/PD-L1	44 (62.9) 26 (37.1)
**Metastatic Sites Number** ≥ 2 < 2	33 (47.1) 37 (52.9)
**Lymph Node Metastasis** No Yes	9 (12.9) 61 (87.1)
**Pleural Metastasis** No Yes	47 (67.1) 23 (32.9)
**Adrenal Metastasis** No Yes	63 (90.0) 7 (10.0)
**Lung Metastasis** No Yes	28 (40.0) 42 (60.0)
**Brain metastasis** No Yes	57 (81.4) 13 (18.6)
**Liver metastasis** No Yes	58 (82.9) 12 (17.1)
**Bone metastasis** No Yes	50 (71.4) 20 (28.6)
**Baseline steroids** No Yes	36 (51.4) 34 (48.6)
**Baseline opioids** No Yes	54 (77.1) 16 (22.9)
**Outcome** Responders Non-Responders	18 (25.7) 52 (74.3)

### Differential abundance of salivary taxa associated with ICIs response

We first assessed microbial diversity to explore broad community-level differences between responders and non-responders. Although no significant differences in α- or β-diversity were observed between responders and non-responders, this does not preclude the presence of meaningful compositional shifts within specific bacterial taxa that may correlate with ICI response (Supplementary Fig. 3–4).

**Fig. 1:**
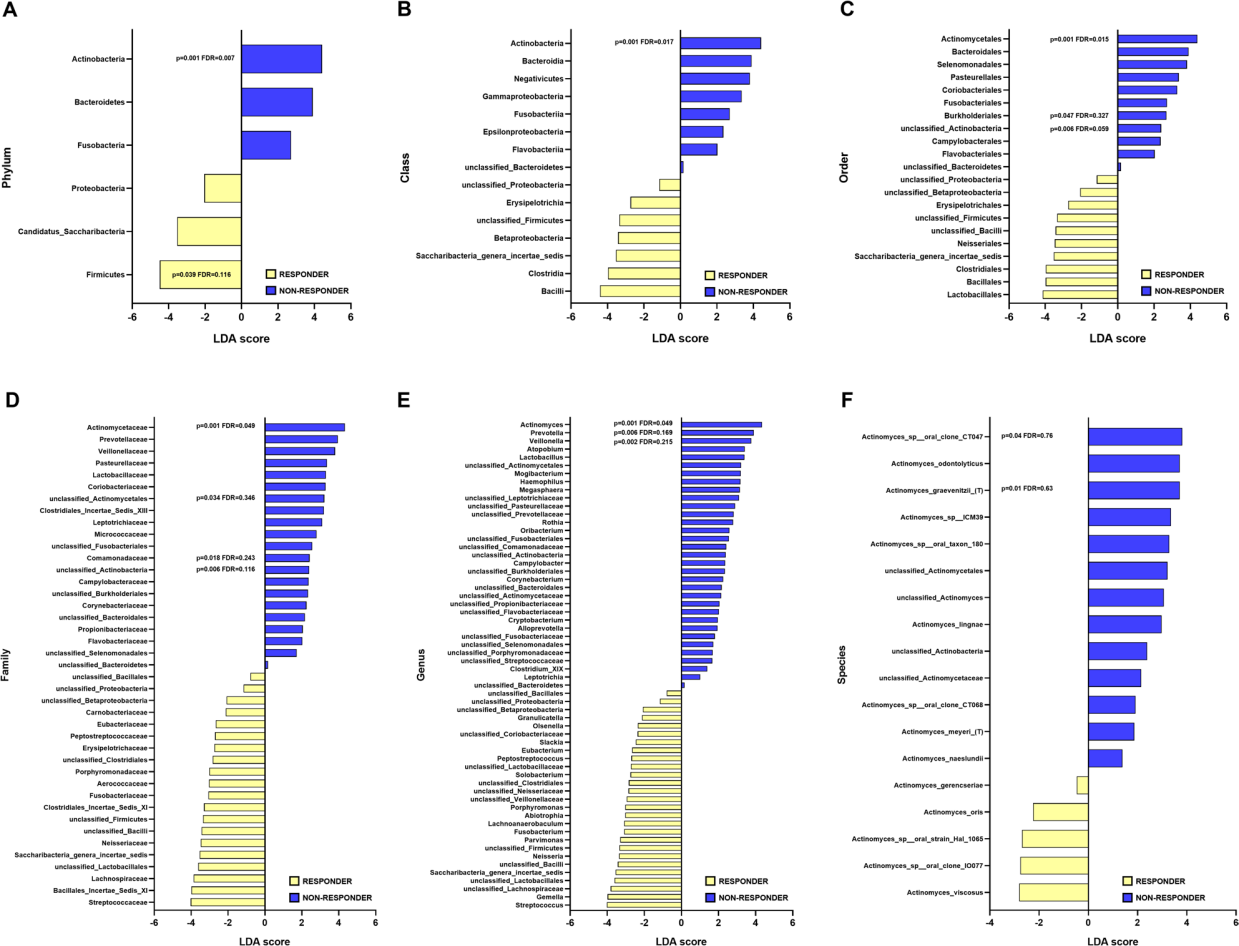
Differential abundance of Actinobacteria across taxonomic ranks. LEfSe analysis comparing responders and non-responders at (**A**) phylum, (**B**) class, (**C**) order, (**D**) family, (**E**) genus and (**F**) Actinomyces species

To identify specific taxonomic groups potentially associated with treatment response, we performed a LEfSe analysis. Notably, Actinobacteria abundance was consistently and significantly higher across all taxonomic levels, from phylum to genus, in non-responders (Fig. 1). This association remained robust after multiple comparison correction (FDR), reinforcing its potential biological relevance. Specifically, Actinobacteria at the phylum level showed increased abundance in non-responders (*p* = 0.001, FDR = 0.007) (Fig. 1A), and this pattern was consistent for the class Actinobacteria (*p* = 0.001, FDR = 0.018) (Fig. 1B), order Actinomycetales (*p* = 0.001, FDR = 0.017) (Fig. 1C), and family Actinomycetaceae (*p* = 0.001, FDR = 0.049) (Fig. 1D). At the genus level, *Actinomyces* emerged as the lowest taxonomic rank to maintain statistical significance after FDR correction (*p* = 0.001, FDR = 0.049), as depicted in Fig. 1E. Among the species within the *Actinomyces* subgroup, *Actinomyces graevenitzii* also showed a greater discriminatory power between responders and non-responders (*p* = 0.012); however, the association lost statistical significance after FDR adjustment (FDR = 0.63) (Fig. 1F).

**Fig. 2:**
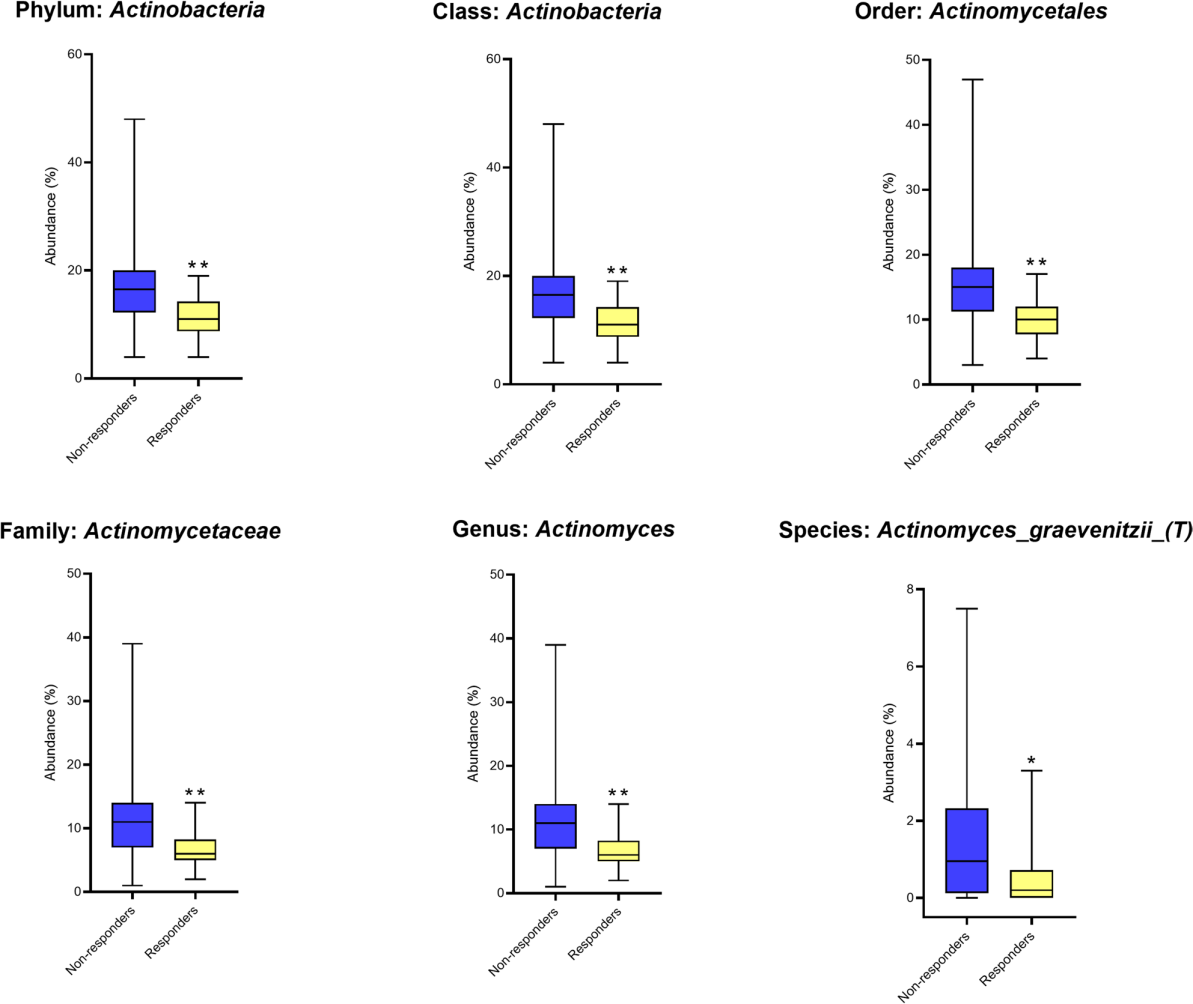
Single-factor analysis of differential taxa. Boxplots from Mann–Whitney/Kruskal–Wallis tests showing abundances in non-responders for phylum Actinobacteria, class Actinobacteria, order Actinomycetales, family Actinomycetaceae, genus Actinomyces and species Actinomyces graevenitzii

Similar results were obtained by single-factor analyses using Mann-Whitney and Kruskal-Wallis tests, with FDR correction applied. Significantly higher abundances of Actinobacteria at the phylum (*p* = 0.001, FDR = 0.005), class (*p* = 0.001, FDR = 0.013), order Actinomycetales (*p* = 0.001, FDR = 0.014), family Actinomycetaceae (*p* = 0.001, FDR = 0.040), and genus *Actinomyces* (*p* = 0.001, FDR = 0.049) were observed in non-responders compared to responders (Fig. 2). At the species level, *Actinomyces graevenitzii* showed a significant difference (*p* = 0.012), which did not remain significant after FDR adjustment (FDR = 0.70) (Fig. 2).

### Prognostic value of salivary actinobacteria in survival outcomes

Given the greater biological specificity and clinical relevance of lower taxonomic ranks, we focused our survival analysis primarily at the genus level. The median relative abundance of the genus *Actinomyces* in the whole cohort was 9% (range 1–39%). No significant differences in *Actinomyces* abundance were found across clinical variables such as age, sex, smoking status, ECOG performance status, PD-L1 expression, histology, treatment line, or the presence of metastases, indicating that its distribution was independent of baseline clinical characteristics (Supplementary Table 1). Notably, a higher relative abundance of *Actinomyces*, analyzed as a continuous variable, was significantly associated with worse outcomes, with a HR for PFS of 1.06 (95% CI: 1.01–1.11, *p* = 0.024) and for OS of 1.05 (95% CI: 1.00–1.10, *p* = 0.038) (Supplementary Table 2).

To enhance clinical translatability, ROC curve analyses were conducted to define optimal cut-off values for stratifying patients into high- and low-abundance groups. The ROC-derived optimal threshold for *Actinomyces* abundance to discriminate between responders and non-responders to ICI treatment was 11% (AUC 0.768, sensitivity 0.94, specificity 0.44), classifying patients as “≤11%” (*n* = 46) or “>11%” (*n* = 24) (Supplementary Fig. 5).

**Fig. 3:**
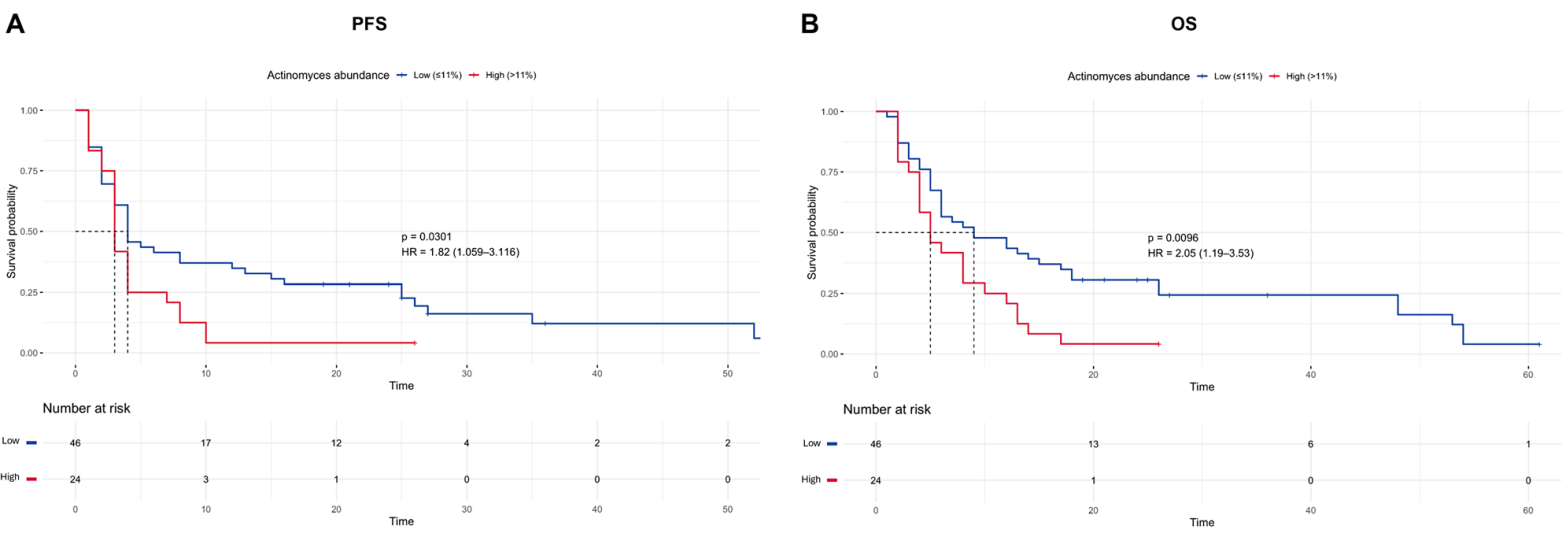
Kaplan–Meier survival curves by Actinomyces abundance. Progression-free survival (**A**) and overall survival (**B**) stratified by optimal genus-level cutoff of Actinomyces abundance (≤ 11% vs. > 11%)

Kaplan-Meier analysis showed that patients with *Actinomyces* abundance > 11% had significantly shorter PFS and OS compared to those with ≤ 11% (Fig. 3). The median PFS was 4 months (95% CI: 3–13) for the ≤ 11% group and 3 months (95% CI: 3–4) for the > 11% group (log-rank *p* = 0.03) (Fig. 3A). Median OS was 9 months (95% CI: 6–18) for patients with lower abundance, compared to 5 months (95% CI: 4–10) for those with higher levels (log-rank *p* = 0.009) (Fig. 3B).

**Fig. 4:**
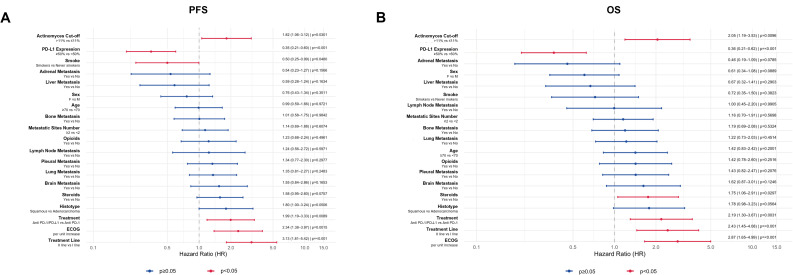
Univariate Cox proportional-hazards analysis. Forest plots depicting hazard ratios (HRs) and 95% confidence intervals for PFS and OS. Actinomyces > 11% and key clinical variables (ECOG status, treatment line, steroids, smoking, PD-L1 expression) are shown with corresponding p values

On univariate analysis (Fig. 4), *Actinomyces* abundance > 11% was significantly associated with worse outcomes, both in terms of PFS (HR = 1.82, 95% CI: 1.06–3.12, *p* = 0.030) and OS (HR = 2.05, 95% CI: 1.19–3.53, *p* = 0.009). Worse ECOG performance status was significantly associated with shorter PFS (HR = 2.34, 95% CI: 1.38–3.97, *p* = 0.001) and OS (HR = 2.87, 95% CI: 1.65–4.99, *p* ≤ 0.001). Second-line treatment was also negatively associated with both PFS (HR = 3.13, 95% CI: 1.81–5.42, *p* ≤ 0.001) and OS (HR = 2.43, 95% CI: 1.45–4.08, *p* ≤ 0.001). Steroid use was significantly associated only with shorter OS (HR = 1.75, 95% CI: 1.06–2.91, *p* = 0.030), while smoking status (current/former vs. never) was associated only with better PFS (HR = 0.50, 95% CI: 0.25–0.99, *p* = 0.048). High PD-L1 expression (≥ 50%) correlated with improved survival, both for PFS (HR = 0.35, 95% CI: 0.21–0.60, *p* ≤ 0.001) and OS (HR = 0.36, 95% CI: 0.21–0.62, *p* ≤ 0.001).

To objectively select the most robust set of predictors for the multivariable model, we applied LASSO penalized Cox regression rather than simply including all variables with univariate significance. As shown in Supplementary Fig. 6–7, LASSO selected the following 11 variables: *Actinomyces* cut-off, ECOG, treatment line, steroids, opioids, smoking status, histotype, adrenal, liver, pleural and brain metastases.

**Fig. 5:**
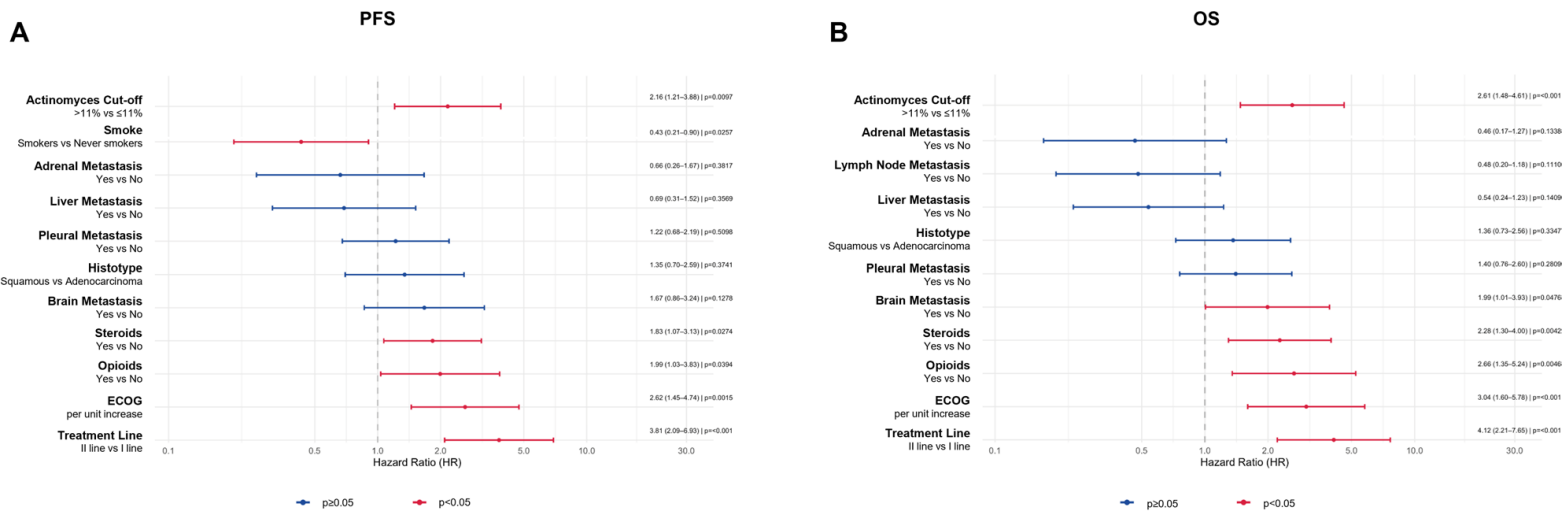
Multivariable Cox proportional-hazards analysis. Adjusted HRs (95% CI) for PFS and OS from the multivariable model including Actinomyces > 11%, ECOG status, treatment line, steroids, opioids, smoking status, histology, and metastatic sites

On multivariate analysis (Fig. 5), *Actinomyces* abundance > 11% remained independently associated with shorter PFS (HR = 2.16, 95% CI: 1.21–3.88, *p* = 0.009) and OS (HR = 2.61, 95% CI: 1.48–4.61, *p* ≤ 0.001). ECOG was a significant independent predictor for both endpoints, with worse scores correlating with reduced PFS (HR = 2.62, 95% CI: 1.45–4.74, *p* = 0.015) and OS (HR = 3.04, 95% CI: 1.60–5.78, *p* ≤ 0.001). Similarly, treatment in second line was associated with worse PFS (HR = 3.81, 95% CI: 2.09–6.93, *p* ≤ 0.001) and OS (HR = 4.12, 95% CI: 2.21–7.65, *p* ≤ 0.001). Steroid use also showed a significant negative impact on both PFS (HR = 1.83, 95% CI: 1.07–3.13, *p* = 0.027) and OS (HR = 2.28, 95% CI: 1.30–4.00, *p* = 0.004). Opioid use was another independent factor associated with worse PFS (HR = 1.99, 95% CI: 1.03–3.83, *p* = 0.039) and OS (HR = 2.66, 95% CI: 1.35–5.24, *p* = 0.004). Smoking status was independently associated only with PFS (HR = 0.43, 95% CI: 0.21–0.90, *p* = 0.025), while the presence of brain metastases was significantly associated only with OS (HR = 1.99, 95% CI: 1.01–3.93, *p* = 0.047).

**Fig. 6:**
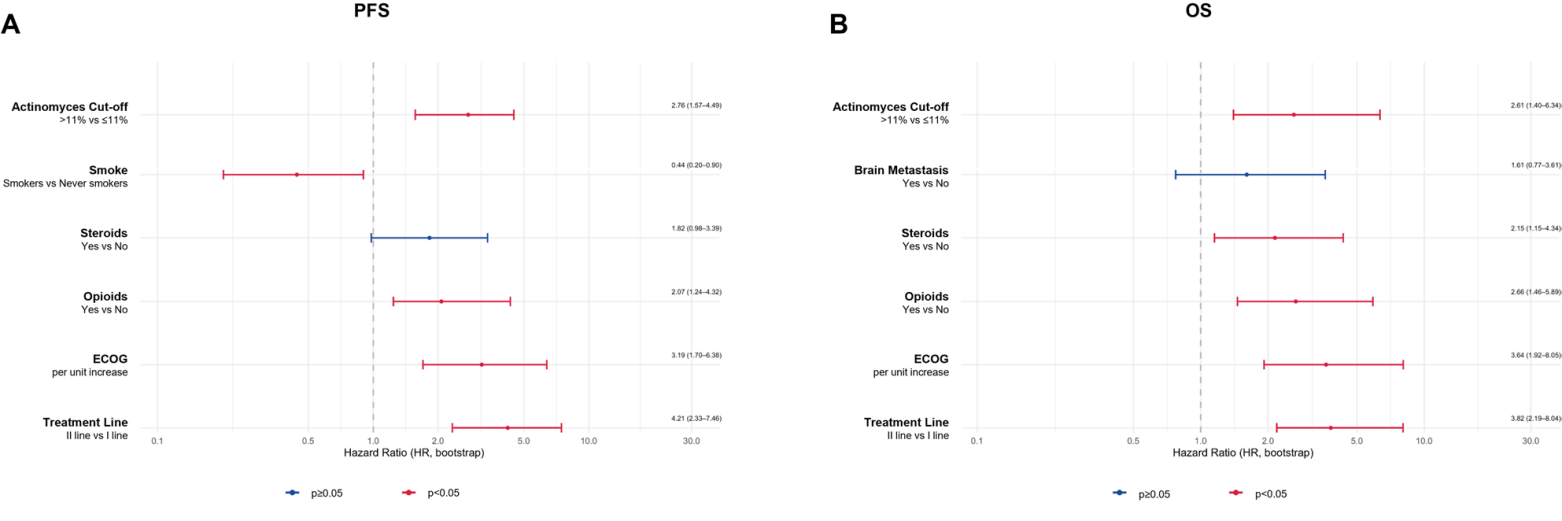
Bootstrap validation of multivariable model. Distribution of HRs across 1,000 bootstrap replicates for PFS and OS

Internal validation using 1,000-fold bootstrap resampling confirmed the robustness of the multivariate Cox model. Several variables consistently emerged as significant predictors of both PFS and OS, with stable effect sizes across endpoints. *Actinomyces* > 11% was associated with worse outcomes, showing a median HR of 2.76 (95% CI: 1.57–4.49) for PFS and 2.61 (95% CI: 1.40–6.34) for OS. ECOG was the strongest clinical predictor, with a median HR of 3.19 (95% CI: 1.70–6.38) for PFS and 3.64 (95% CI: 1.92–8.05) for OS. Second-line treatment (PFS: HR = 4.21, 95% CI: 2.33–7.46; OS: HR = 3.82, 95% CI: 2.19–8.04), and opioid use (PFS: HR = 2.07, 95% CI: 1.24–4.32; OS: HR = 2.66, 95% CI: 1.46–5.89) also retained statistical significance and consistent direction of effect. Moreover, smoking status was associated only with better PFS (HR = 0.44, 95% CI: 0.20–0.90), while steroid use was correlated only with reduced OS (HR = 2.15, 95% CI: 1.15–4.34) (Fig. 6). Overall, the bootstrap confidence intervals closely mirrored those of the original model, reinforcing the internal validity and reliability of the selected prognostic factors.

Collectively, these data provide robust evidence that a higher abundance of Actinobacteria-particularly at the genus level of *Actinomyces*-consistently correlates with poorer survival outcomes, and support its potential as a hypothesis-generating biomarker in patients with advanced NSCLC treated with ICIs.

## Discussion

This study shows that salivary enrichment of *Actinomyces* is independently associated with shorter PFS and OS in NSCLC patients treated with ICIs. These findings expand the scope of microbiome biomarkers in immuno-oncology, indicating that the salivary microbiota, like its intestinal counterpart, may modulate response to checkpoint blockade. Although direct mechanistic data in lung cancer are still emerging, colorectal cancer models have shown that *Actinomyces spp*. activate TLR2/NF-κB signaling in tumor-infiltrating myeloid cells, driving IL-10 and TGF-β production and impairing CD8⁺ T-cell recruitment [12]. Complementary work indicates that short-chain fatty acids such as butyrate and propionate, metabolites of *Actinomyces* fermentation, inhibit dendritic cell maturation and IL-12 secretion, thereby compromising antigen presentation and initial T-cell priming [13]. Together, these immunosuppressive effects prior to therapy may blunt the efficacy of subsequent ICI-induced antitumor responses, offering a plausible biological explanation for the link between *Actinomyces* abundance and poor clinical outcomes. A recent study in hepatocellular carcinoma patients receiving ICIs similarly identified *Actinomyces* as enriched in non-responders [14], supporting the hypothesis that *Actinomyces*-mediated immunosuppression contributes to resistance to checkpoint blockade. Moreover, experimental infection of BALB/c mice with *Nocardia brasiliensis*,a close relative of *Actinomyces*, induces an immunosuppressive milieu characterized by upregulation of the PD-1/PD-L1 axis, expansion of FoxP3⁺ regulatory T cells, and accumulation of myeloid-derived suppressor cells (MDSCs) [15, 16]. Oral inflammatory diseases and key oral pathogens also modulate MDSC function and skew macrophages toward an M2-like phenotype, further attenuating T-cell–mediated immunity [17]. By analogy, chronic oral colonization by *Actinomyces* may recruit similar suppressive circuits within the lung tumor microenvironment, undermining checkpoint blockade efficacy.

*Actinomyces* persistence in the oral cavity is underpinned by its robust biofilm-forming capacity. Microscopic analyses reveal that *Actinomyces* constructs multilayered, protein- and polysaccharide-rich biofilms on tooth surfaces, facilitating adhesion, nutrient trapping, and resistance to host defenses [18]. A comprehensive review of human actinomycoses highlights these bacteria’s ability to invade mucosal tissues and establish chronic, granulomatous lesions, evading immune clearance [19]. Although these studies focus on oral and cutaneous infections, similar biofilm-based mechanisms may enable salivary *Actinomyces* to seed the lung via microaspiration and sustain a tolerogenic microenvironment. Screening studies have identified natural compounds, such as aqueous herbal extracts, with potent activity against oral *Actinomyces*, suggesting potential interventions to disrupt these biofilms [20]. Although highly speculative, these observations raise the hypothesis that, in future studies, targeted oral formulations (e.g., specialized mouthwashes or lozenges) containing biofilm-disrupting agents might be explored as adjuvant strategies to reduce *Actinomyces* burden prior to ICI therapy; at present, however, such interventions remain entirely experimental.

In contrast, Huang et al. [21] reported that in advanced NSCLC patients receiving chemo-immunotherapy, responders exhibited higher *Actinomyces* abundance alongside elevated lysophosphatidylcholines and ceramide-like metabolites associated with improved survival. This discrepancy likely reflects key methodological differences, including the concurrent administration of chemotherapy in their study and the considerably smaller sample size (*n* = 20) compared to our cohort (*n* = 70), which exclusively received ICI monotherapy.

Mechanistic links between the oral niche and pulmonary immunity are in part mediated by microaspiration. Segal et al. showed that enrichment of oral taxa, such as Prevotella and Veillonella, in bronchoalveolar lavage fluid of healthy volunteers associates with a Th17-skewed inflammatory phenotype, suggesting that oral commensals can seed and modulate the lung microenvironment [11]. In NSCLC models, Tsay et al. reported that oral bacteria colonizing the lung activate oncogenic extracellular signal-regulated kinase (ERK) and phosphoinositide 3-kinase (PI3K) pathways in airway epithelial cells [22], further implicating oral–pulmonary microbial crosstalk in tumor progression.

From a translational perspective, quantifying salivary *Actinomyces* may, if validated, complement conventional biomarkers (e.g., PD-L1 expression, tumor mutational burden) to refine patient selection for frontline ICI therapy. In this exploratory cohort, a higher abundance of *Actinomyces* was associated with poorer outcomes, suggesting that pre-treatment salivary profiling could potentially help identify patients at higher risk of non-response, thereby informing closer monitoring or the choice of intensified regimens. However, these potential clinical applications remain hypothetical and require confirmation in larger, independent cohorts before salivary *Actinomyces* can be implemented as a stratification tool. In particular, the ROC-derived 11% cut-off, which yielded high sensitivity but low specificity, is best interpreted as an exploratory, screening-oriented threshold to flag patients at increased risk rather than as a tool capable of reliably discriminating responders and non-responders on an individual basis.

Our study has limitations. The cohort size is modest with a predominance of non-responders, which may introduce bias and limit generalizability, and the number of events relative to the covariates included in the multivariable models raises the possibility of overfitting despite the use of LASSO selection and bootstrap internal validation. Baseline-only sampling does not capture microbiota dynamics during therapy, and potential confounders, such as oral health status or comorbidities, were not systematically assessed. Importantly, we did not systematically collect data on oral hygiene, periodontal status, or related behaviors (e.g., frequency of dental care, use of mouthwashes), which may influence salivary microbiota composition and act as residual confounders. No structured information on dental status, recent dental procedures, or clinical signs of oral inflammation was available beyond routine clinical documentation. Poor oral hygiene (e.g., periodontitis) might elevate Actinomyces levels and independently influence immune competence, representing a potential source of bias. Similarly, although use of systemic antibiotics within 20 days before sampling was an exclusion criterion, chronic antibiotic or probiotic intake beyond this window was not prospectively recorded and could not be evaluated in sensitivity analyses. The single-center design further limits external validity. Moreover, the modest sample size within PD-L1 expression subgroups means that our study is not powered to robustly evaluate interaction between PD-L1 status and Actinomyces abundance; such analyses will require larger, multicentre cohorts. To address these issues, future studies will prospectively collect detailed oral-health and behavioral covariates, include multi-timepoint sampling of saliva and stool during ICI therapy, and seek external validation in larger, independent cohorts.

## Conclusion

Our data, supported by the literature, indicate that the salivary microbiota, particularly *Actinomyces* abundance, is strongly associated with clinical outcomes in NSCLC patients treated with ICIs. Integrating multi-compartment microbiome profiling with host and tumor biomarkers may yield composite indices capable of optimizing patient stratification and guiding microbiome-targeted adjuvant strategies. At present, salivary Actinomyces should be regarded as a promising, hypothesis-generating biomarker that warrants further validation rather than a ready-to-use clinical tool. The salivary microbiome thus emerges as a promising, accessible biomarker and therapeutic target for enhancing ICI responses. Further translational and mechanistic studies are needed to clarify its role in cancer immunotherapy and develop clinical interventions to modulate the salivary microbiota.

## Supplementary Information

Below is the link to the electronic supplementary material.

Supplementary Material 1

## Data Availability

All data relevant to the study are included in the article or uploaded as supplementary information.

## Abbreviations

ICIs: Immune Checkpoint Inhibitors
NSCLC: Non-Small Cell Lung Cancer
PFS: Progression-Free Survival
OS: Overall Survival
PD-L1: Programmed Death-Ligand 1
ECOG: Eastern Cooperative Oncology Group
TSS: Total sum scaling
PCoA: Principal Coordinates Analysis
PERMANOVA: Permutational Multivariate Analysis of Variance
LEfSe: Discriminant Analysis Effect Size
LDA: Linear Discriminant Analysis
FDR: False Discovery Rate
ROC: Receiver Operating Characteristic
AUC: Area Under the Curve
CI: confidence intervals
HR: hazard ratios
LASSO: Least Absolute Shrinkage and Selection Operator
MDSCs: Myeloid-Derived Suppressor Cells
ERK: Extracellular signal-Regulated Kinase
PI3K: Phosphoinositide 3-Kinase
