# Supplementary material for: Salivary microbial signature highlighting actinomyces as a predictor of immune-checkpoint inhibitor monotherapy response in advanced non–small cell lung cancer
Source: J Transl Med. 2026 Jan 17;24:180. doi: 10.1186/s12967-025-07570-4 (PMC12895580; doi:10.1186/s12967-025-07570-4)
Supplement: Supplementary file 1 — Supplementary Material 1 [file 12967_2025_7570_MOESM1_ESM.pdf]

Supplementary Figures

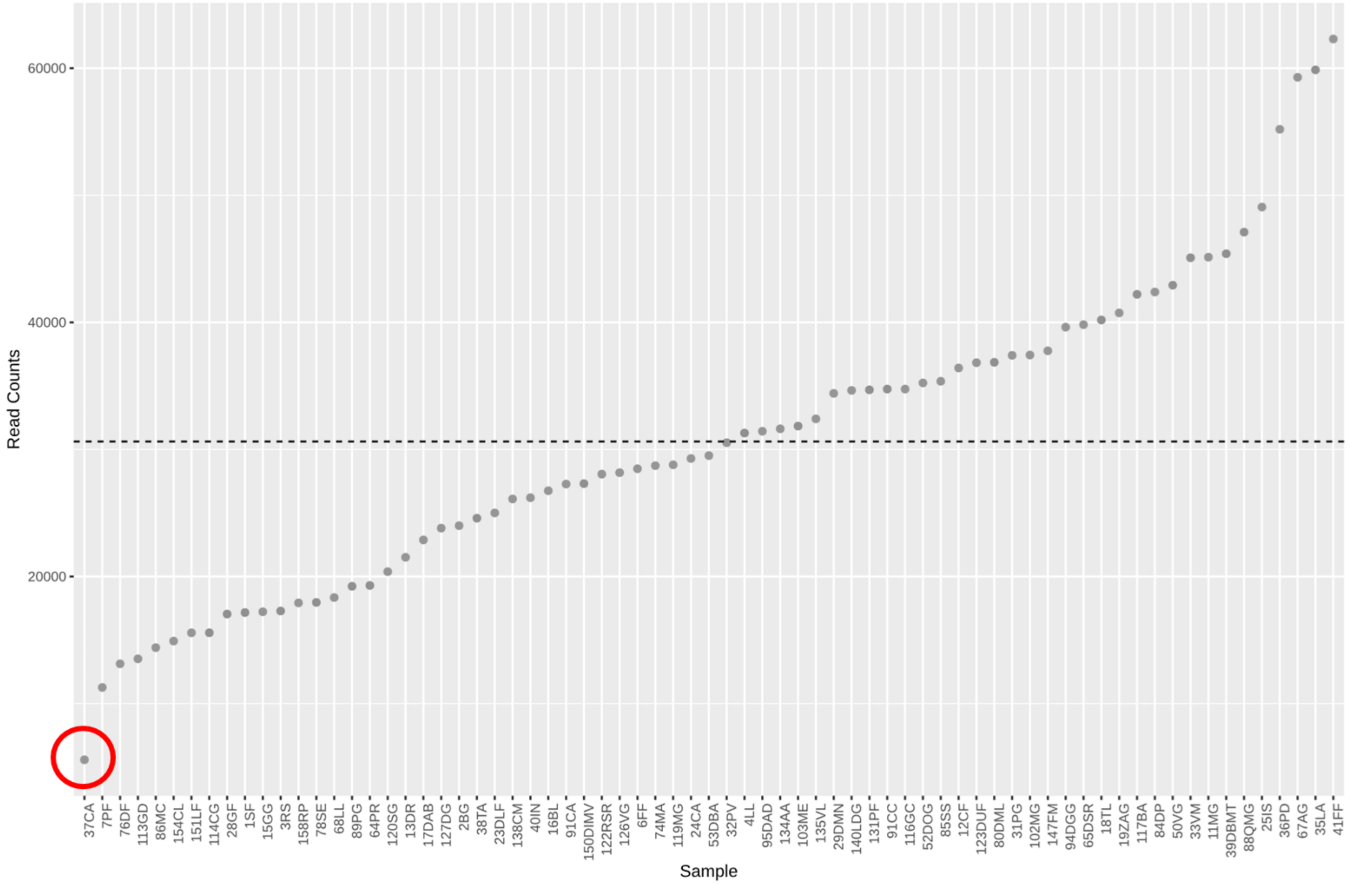

Supplementary Figure 1. Diagram showing enrollment of 71 patients, exclusion of one sample due to low library size, and final inclusion of 70 saliva specimens for 16S rRNA sequencing and downstream analyses.

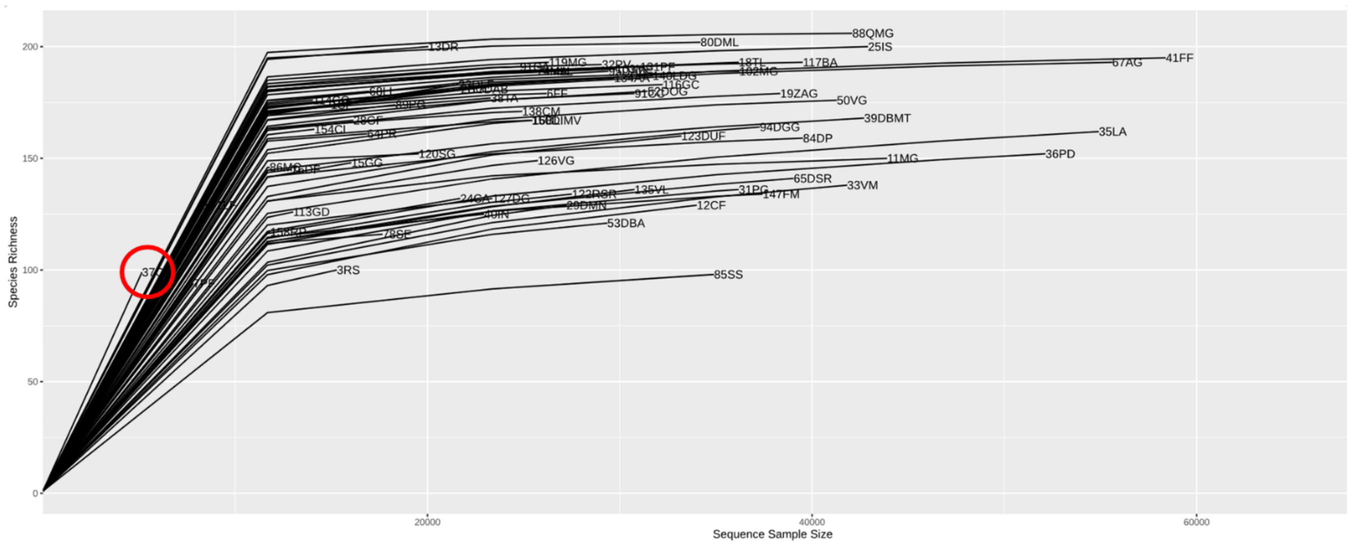

Supplementary Figure 2. Per-sample read counts before and after filtering, and distribution of OTU counts across samples following clustering at ≥ 97% sequence identity.

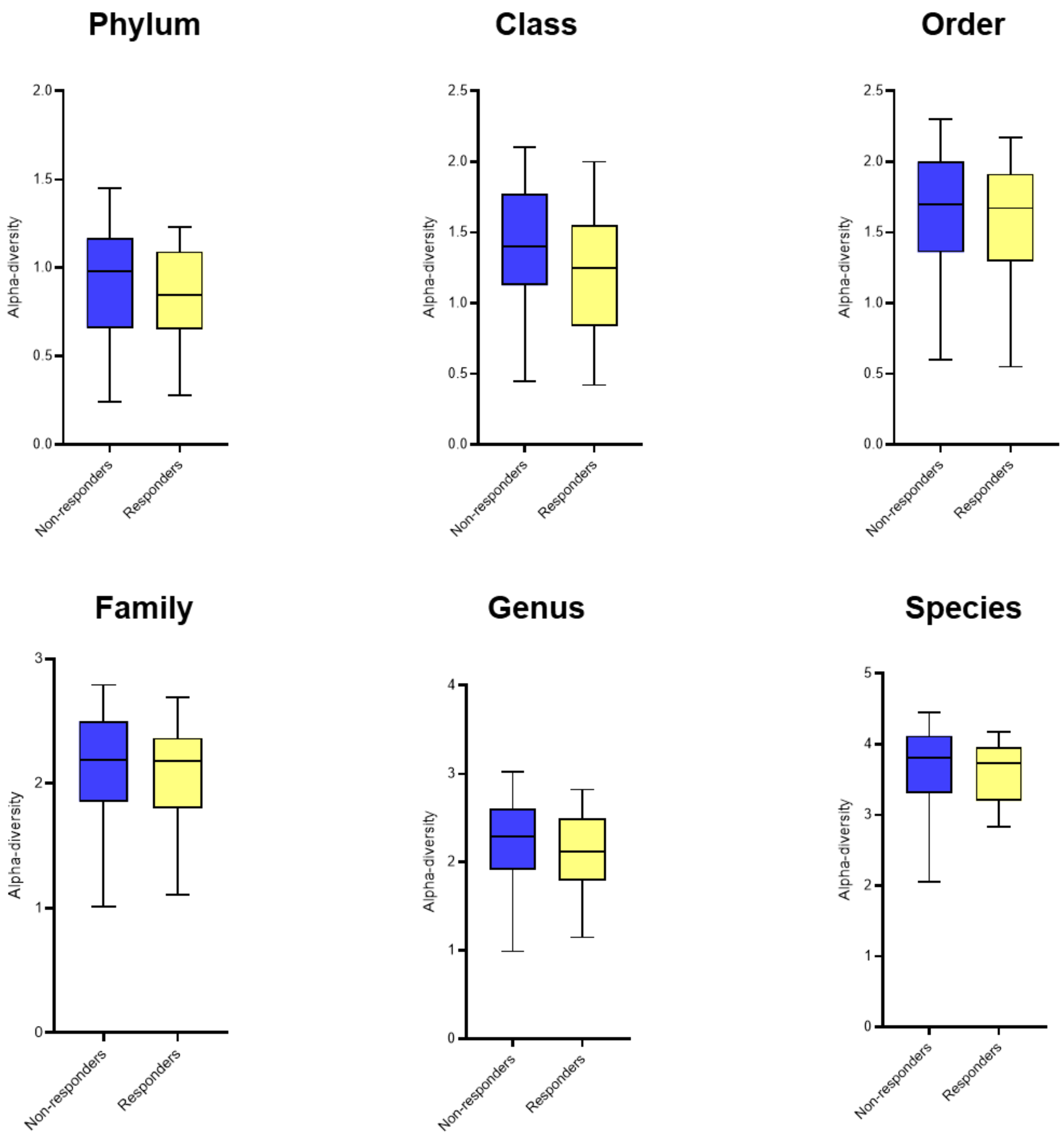

Supplementary Figure 3. α-diversity comparison between responders and non-responders**.**

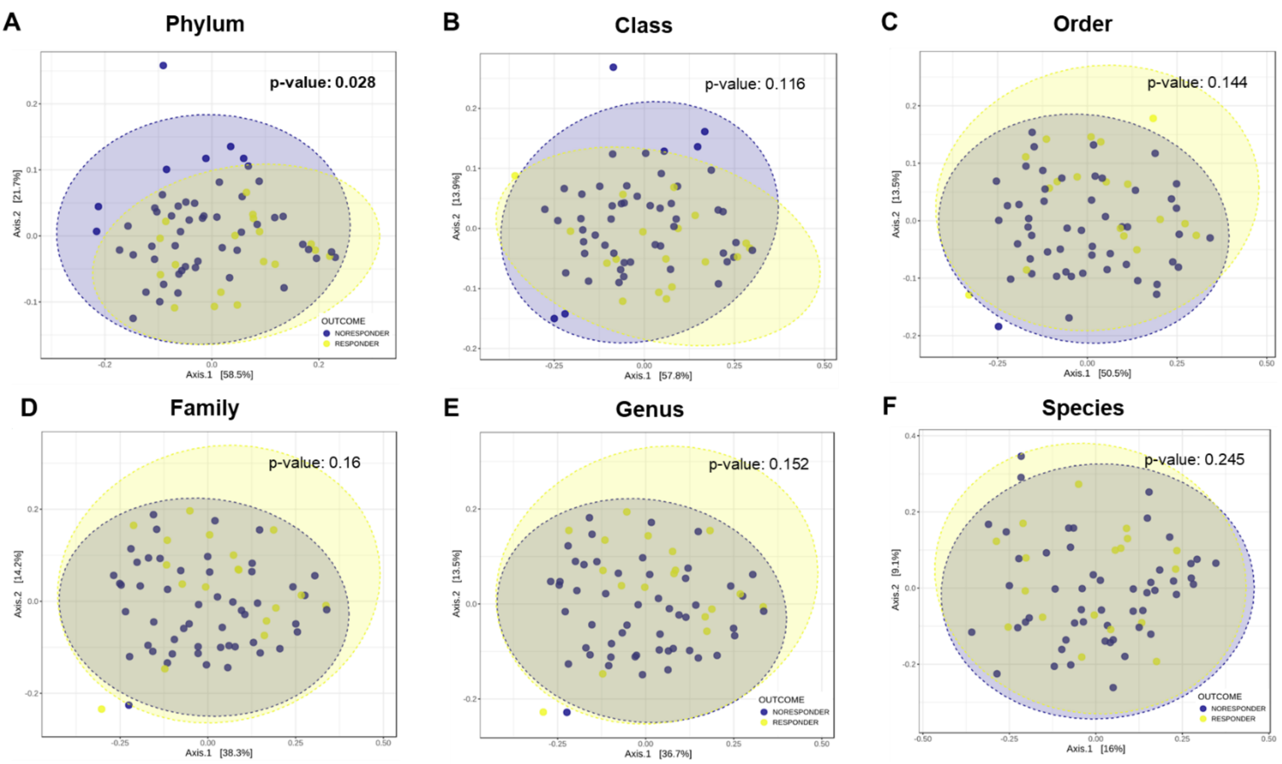

Supplementary Figure 4. β-diversity ordination by PCoA.

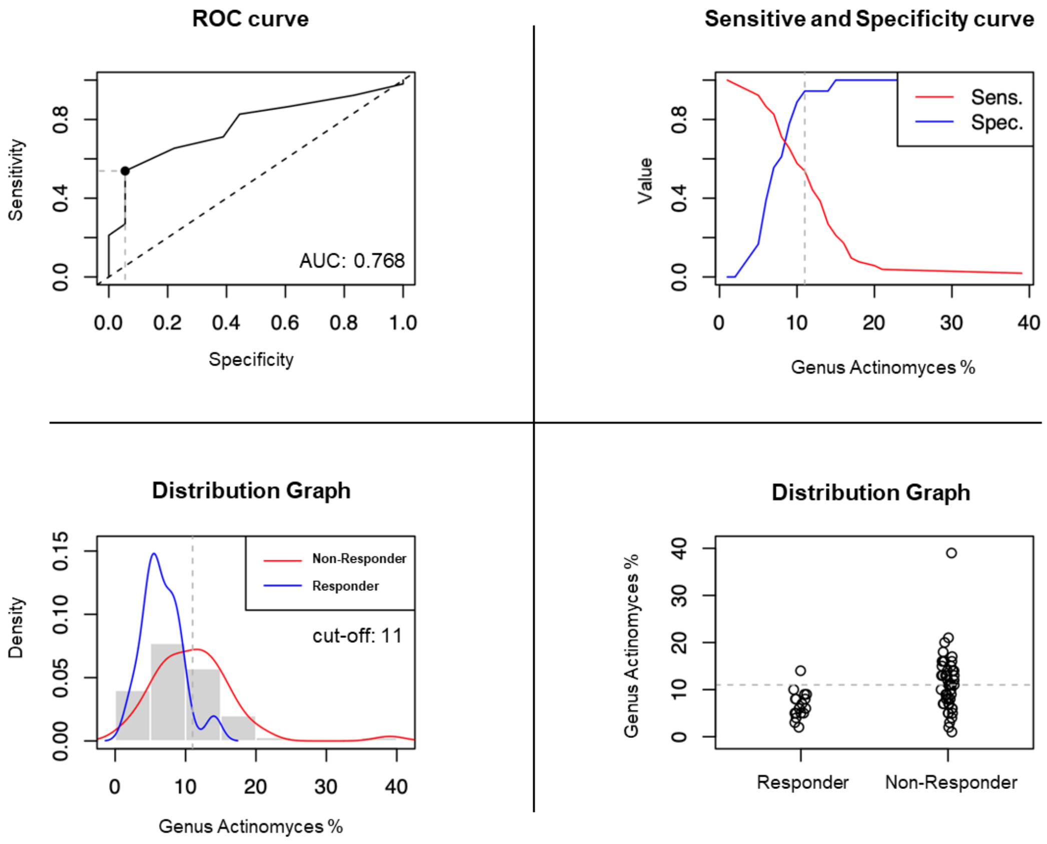

Supplementary Figure 5. Receiver operating characteristic (ROC) curve for *Actinomyces* cut-off

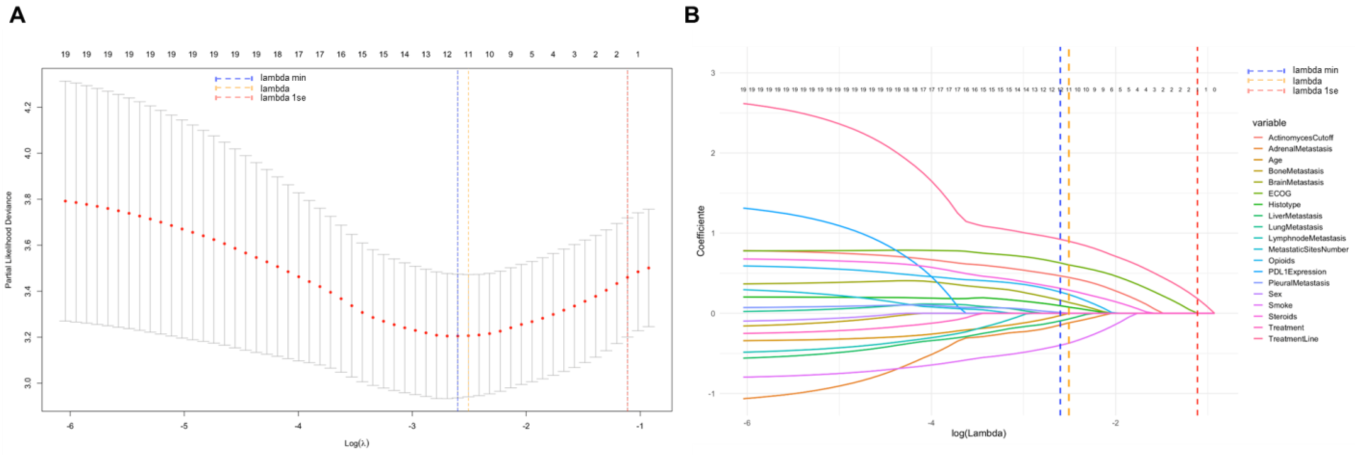

Supplementary Figure 6. LASSO variable selection path for PFS model. Plot of coefficient trajectories across increasing penalization (λ) and selection of variables at the chosen λ based on balance between model complexity and events.

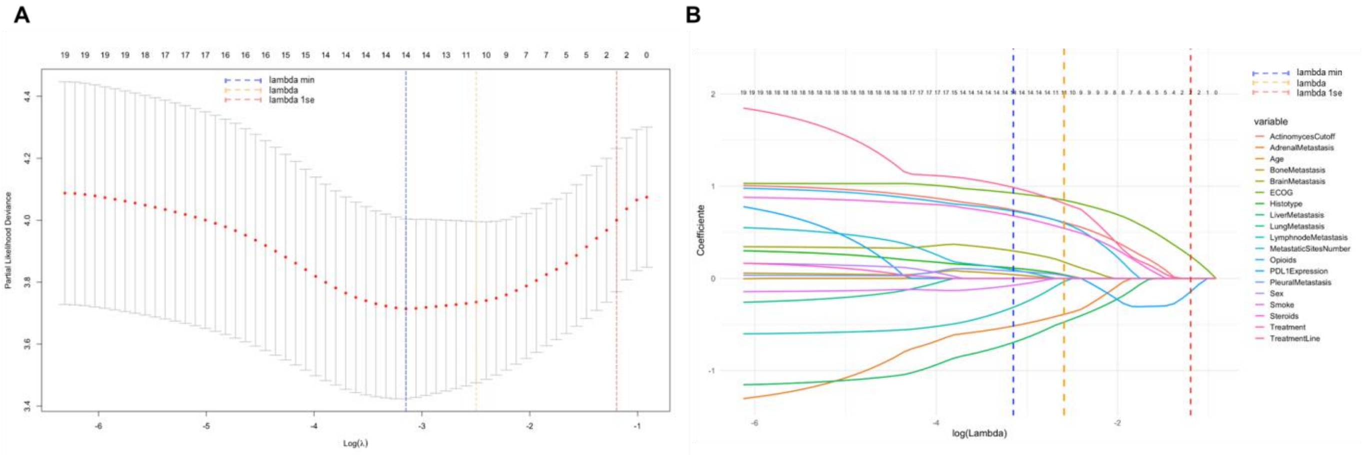

Supplementary Figure 7. LASSO variable selection path for OS model. Coefficient shrinkage trajectories and selected predictors at the optimal λ for overall survival.

Actinomyces genus %		
**Feature (N° of pts)**	**Median (range)**	**p-value**
**Age**		
≥70 (28)	9 (3 - 17)	p = 0.609
<70 (42)	9 (1 - 39)	
**Sex**		
Female (21)	8 (2 - 18)	p = 0.227
Male (49)	9 (1 - 39)	
**Smoke**		
Never (10)	9.5 (5 - 18)	p = 0.774
Former/Current (60)	66 (1 - 39)	
**ECOG-PS**		
0 (30)	9.5 (2 - 21)	p = 0.486
1 (40)	8.5 (1 - 39)	
**PD-L1 Expression**		
≥ 50% (39)	9 (5 - 15)	p = 0.987
1-49% (14)	9.5 (2 - 16)	
< 1% (17)	9 (1 – 39)	
**Histology**		
Adenocarcinoma (54)	9 (2 - 39)	p = 0.653
Squamous (16)	9 (1 - 14)	
**Treatment Line**		
First (35)	9 (1 - 39)	p = 0.625
Non-first (35)	9 (2 - 16)	
**Treatment Type**		
Anti PD-1 (44)	9 (1 - 39)	p = 0.351
Anti PD-1/PD-L1 (26)	8 (2 - 16)	
**Metastatic Sites Number**		
≥ 2 (33)	8 (2 - 39)	p = 0.766
< 2 (37)	10 (1 - 20)	
**Lymph Node Metastasis**		
No (9)	10 (1 - 16)	p = 0.451
Yes (61)	9 (2 - 39)	
**Pleural Metastasis**		
No (47)	9 (1 - 39)	p = 0.872
Yes (23)	11 (3 - 21)	
**Adrenal Metastasis**		
No (63)	9 (1 - 39)	p = 0.893
Yes (7)	9 (5 - 14)	
**Lung Metastasis**		
No (28)	10 (3 - 20)	p = 0.889
Yes (42)	8 (1 - 39)	
**Brain Metastases**		
No (57)	9 (2 - 39)	p = 0.491
Yes (13)	9 (1 - 20)	
**Liver Metastases**		
No (58)	9.5 (1 - 39)	p = 0.077
Yes (12)	7 (2 - 14)	
**Bone Metastases**		
No (50)	9 (1 - 21)	p = 0.839
Yes (20)	9 (2 - 39)	
**Baseline Opioids**		
No (54)	10 (2 - 39)	p = 0.139
Yes (16)	9 (1 - 14)	
**Baseline Steroids**		
No (36)	9 (2 - 20)	p = 0.454
Yes (34)	9.5 (1 - 39)	

Supplementary Table 1. Univariate associations of Actinomyces abundance with clinical variables

A **Cox Table – Univariable - PFS**

	Mean (SD)	HR
**Phylum -** ***Actinobacteria***	15.4 (6.9)	1.05 (1.01-1.09, p=0.008)
**Class -** ***Actinobacteria***	15.4 (6.9)	1.05 (1.01-1.09, p=0.008)
**Order -** ***Anctinomycetales***	13.7 (6.4)	1.06 (1.02-1.11, p=0.004)
**Family -** ***Actinomycetaceae***	10.1 (5.7)	1.06 (1.01-1.11, p=0.024)
**Genus -** ***Actinomyces***	10.0 (5.6)	1.06 (1.01-1.11, p=0.024)

B **Cox Table – Univariable - OS**

	Mean (SD)	HR
**Phylum -** ***Actinobacteria***	15.4 (6.9)	1.05 (1.01-1.09, p=0.016)
**Class -** ***Actinobacteria***	15.4 (6.9)	1.05 (1.01-1.09, p=0.016)
**Order -** ***Anctinomycetales***	13.7 (6.4)	1.05 (1.01-1.10, p=0.011)
**Family -** ***Actinomycetaceae***	10.1 (5.7)	1.06 (1.00-1.10, p=0.039)
**Genus-** ***Actinomyces***	10.0 (5.6)	1.05 (1.00-1.10, p=0.038)

Supplementary Table 2. Univariate Cox regression for Actinomyces as continuous variable
